# Supplementary material for: Developing a multivariate prediction model of antibody features associated with protection of malaria-infected pregnant women from placental malaria
Source: eLife. 2021 Jun 29;10:e65776. doi: 10.7554/eLife.65776 (PMC8241440; doi:10.7554/eLife.65776)
Supplement: Figure 3—source data 1. — A PLSDA model with two components and using the six selected antibody features can be formed for prediction of the group of the pregnant women. The estimated loading factors of the model for the two components (shown in Figure 3) are listed here. The softmax technique was used to normalize the scores for each class (placental malaria and non-placental infection) that works as the probability of an observation belonging to a certain class (Kuhn, 2008). The predicted class is the one with the largest model prediction or, equivalently, the largest class probability. [file elife-65776-fig3-data1.docx]

**Figure 3-source data 1: PLSDA prediction model.**

| **Antibody feature** | **Loading on component 1** | **Loading on component 2** |
| --- | --- | --- |
| IgG3.DBL2(ID1-ID2a).FCR3 | 0.45 | 0.56 |
| THP1.Phago.CS2 | 0.52 | -0.52 |
| Neutrophil.Phago.CS2 | 0.36 | 0.2 |
| CSA.Binding.Inhibition.FCR3 | 0.29 | 0.23 |
| IgA2.DBL2.1010 | 0.41 | 0.21 |
| IgG3.3D7 | 0.38 | -0.53 |
