# Supplementary material for: Developing a multivariate prediction model of antibody features associated with protection of malaria-infected pregnant women from placental malaria
Source: eLife. 2021 Jun 29;10:e65776. doi: 10.7554/eLife.65776 (PMC8241440; doi:10.7554/eLife.65776)
Supplement: Supplementary file 1. [file elife-65776-supp1.docx]

**Supplementary File 1**

**Table: Recombinant VAR2CSA DBL Domain Proteins Used to Measure Antibody Features**

| Label | Assay^a^ | VAR2CSA domain | Isolate (I) or line | Expression System | Lot number | Reference and Source^b^ |
| --- | --- | --- | --- | --- | --- | --- |
| DBL1-7G8 | 1 | DBL1 | 7G8 | *Pichia pastoris* | MV-1398 | *1 (Avril et al., 2011)* |
| DBL2(ID1-ID2a)-FCR3 | 1 | DBL2 | FCR3 | *Escherichia coli* | MV1942 | *1 (Doritchamou et al., 2016)* |
| DBL2(ID1-ID2a)-FCR3 | 2, 3, 4 | DBL2 | FCR3 | *Drosophila* Schneider-2 cell |  | *(Mordmuller et al., 2019)* |
| DBL2-isolate | 1 | DBL2 | 1010 | *E. coli* | MV 1940 | *1 (Doritchamou et al., 2016)* |
| DBL3- FCR3 | 1 | DBL3 | FCR3 | *Drosophila* Schneider-2 cell | MP1028 | *(Nielsen et al., 2009)* |
| DBL3- 7G8 | 1, 2, 3, 4 | DBL3 | 7G8 | *P. pastoris* | MV-1914 | *1 (Avril et al., 2011)* |
| DB4-FCR3 | 1 | DBL4 | FCR3 | *E. coli* | MP2369 | *(Fried et al., 2013)* |
| DBL4-isolate | 1 | DBL4 | I 0711 | *E. coli* | MV1700 | *1 (Doritchamou et al., 2016)* |
| DBL5-3D7 | 1 | DBL5 | 3D7 | *P. pastoris* | 1218 | *(Avril et al., 2011)* |
| DBL5-7G8 | 3 | DBL5 | 7G8 | *P. pastoris* | 1269 | *(Avril et al., 2011)* |
| DBL5-isolate | 1, 3, 4 | DBL5 | I 0466 | *Escherichia coli* | MV 1749 | *1 (Doritchamou et al., 2016)* |
| DBL6-IT4 | 1 | DBL6 | IT4 | *P. pastoris* | MV-1137 | *(Avril et al., 2011)* |
| a multiplex, 1; NK cell assays, 2; neutrophil assays, 3; monocyte assays, 4  b gifted by 1, Patrick Duffy & David Narum;  DBL Domain, duffy binding like domain; ID, interdomain region | | | | | | |
