# Supplementary material for: Developing a multivariate prediction model of antibody features associated with protection of malaria-infected pregnant women from placental malaria
Source: eLife. 2021 Jun 29;10:e65776. doi: 10.7554/eLife.65776 (PMC8241440; doi:10.7554/eLife.65776)
Supplement: Supplementary file 2. [file elife-65776-supp2.docx]

| **Supplementary File 2 Table: Univariate analysis non-infected v placental malaria and non-placental infection v placental malaria** | | | | | |
| --- | --- | --- | --- | --- | --- |
|  | Non-infected | Placental malaria | Non-placental infection | Placental malaria v Non-infected^a^ | Placental malaria v Non-placental infection^a^ |
| Antibody feature | mean (SD) | mean (SD) | mean (SD) | p value | p value |
| IgG1.CS2 | -0.07 (0.95) | -0.18 (0.78) | 0.46 (1.30) | 0.5432 | 0.0267 |
| IgG2.CS2 | -0.11 (0.78) | -0.01 (0.87) | 0.23 (1.48) | 0.5358 | 0.451 |
| IgG3.CS2 | -0.15 (0.62) | -0.18 (0.99) | 0.62 (1.33) | 0.8522 | 0.0083 |
| IgG4.CS2 | -0.17 (0.80) | -0.10 (0.84) | 0.49 (1.41) | 0.6496 | 0.0555 |
| IgG.CS2 | -0.01 (1.04) | -0.14 (0.76) | 0.28 (1.27) | 0.4615 | 0.1185 |
| IgG1.3D7 | 0.06 (1.03) | -0.24 (0.58) | 0.35 (1.40) | 0.0763 | 0.0448 |
| IgG2.3D7 | 0.04 (0.99) | -0.12 (0.99) | 0.16 (1.05) | 0.426 | 0.2617 |
| IgG3.3D7 | -0.15 (0.96) | -0.14 (0.85) | 0.55 (1.17) | 0.9541 | 0.0094 |
| IgG4.3D7 | 0.10 (1.07) | -0.19 (0.85) | 0.17 (1.11) | 0.1434 | 0.1499 |
| IgG.3D7 | -0.06 (0.98) | -0.10 (0.87) | 0.31 (1.22) | 0.83 | 0.1298 |
| THP1.Phago.CS2 | -0.07 (0.98) | -0.29 (0.81) | 0.66 (1.10) | 0.2193 | 0.0003 |
| THP1.Phago.IgG.CS2 | -0.01 (1.01) | -0.07 (0.85) | 0.16 (1.24) | 0.731 | 0.3867 |
| THP1.Phago.3D7 | -0.09 (1.14) | -0.07 (0.85) | 0.31 (0.96) | 0.9124 | 0.0892 |
| THP1.Phago.DBL2 (ID1-ID2a).FCR3 | -0.02 (0.99) | -0.01 (1.01) | 0.04 (1.05) | 0.9702 | 0.8359 |
| THP1.Phago.DBL3.7G8 | 0.07 (1.00) | -0.11 (0.91) | 0.07 (1.18) | 0.3468 | 0.5045 |
| THP1.Phago.DBL5.0466 | -0.08 (1.01) | 0.12 (0.95) | -0.09 (1.08) | 0.3082 | 0.3984 |
| Monocyte.Phago.CS2 | -0.03 (0.89) | -0.18 (1.04) | 0.39 (1.04) | 0.454 | 0.0257 |
| Monocyte.Phago.3D7 | -0.30 (1.23) | 0.03 (0.74) | 0.51 (0.71) | 0.1093 | 0.0064 |
| Monocyte.Phago.DBL2(ID1-ID2a).FCR3 | 0.04 (0.93) | -0.03 (0.96) | -0.02 (1.21) | 0.7223 | 0.9786 |
| Monocyte.Phago.DBL3.7G8 | 0.01 (0.90) | -0.07 (1.08) | 0.11 (1.05) | 0.7147 | 0.4765 |
| Monocyte.Phago.DBL5.7G8 | -0.03 (0.94) | -0.07 (1.08) | 0.17 (0.95) | 0.8441 | 0.3159 |
| Neutrophil.ROS.DBL2.(ID1-ID2a).FCR3 | -0.03 (1.07) | 0.04 (0.97) | -0.01 (0.95) | 0.7288 | 0.8535 |
| Neutrophil.ROS.DBL3.7G8 | 0.03 (0.95) | -0.13 (0.98) | 0.19 (1.12) | 0.3965 | 0.213 |
| Neutrophil.ROS.DBL5.7G8 | -0.12 (0.77) | 0.06 (1.23) | 0.11 (0.90) | 0.3661 | 0.8646 |
| Neutrophil.ROS.CS2 | -0.08 (1.09) | -0.03 (1.00) | 0.20 (0.81) | 0.8046 | 0.27 |
| Neutrophil.Phago.DBL2(ID1-ID2a).FCR3 | -0.08 (0.92) | -0.13 (1.04) | 0.39 (1.00) | 0.7963 | 0.0366 |
| Neutrophil.Phago.DBL3.7G8 | -0.12 (0.97) | -0.04 (1.05) | 0.31 (0.94) | 0.6969 | 0.1305 |
| Neutrophil.Phago.DBL5.7G8 | -0.12 (0.99) | -0.03 (1.02) | 0.27 (0.96) | 0.6497 | 0.2097 |
| Neutrophil.Phago.CS2 | -0.02 (0.99) | -0.22 (1.11) | 0.44 (0.62) | 0.3533 | 0.0012 |
| CSA.Binding.Inhibition.NF54 | 0.13 (0.39) | -0.01 (0.43) | -0.24 (2.04) | 0.0974 | 0.5767 |
| CSA.Binding.Inhibition.FCR3 | 0.14 (0.65) | -0.32 (1.37) | 0.31 (0.46) | 0.0397 | 0.0052 |
| NK.CD107a.IgG.DBL2(ID1-ID2a).FCR3 | NA (NA) | -0.07 (0.84) | 0.13 (1.25) | NA | 0.4566 |
| NK.IFN.IgG.DBL2(ID1-ID2a).FCR3 | NA (NA) | -0.16 (0.83) | 0.30 (1.21) | NA | 0.0817 |
| NK.TNF.IgG.DBL2(ID1-ID2a).FCR3 | NA (NA) | -0.01 (0.93) | 0.01 (1.13) | NA | 0.944 |
| NK.CD107a.IgG.DBL3.7G8 | 0.03 (1.02) | -0.18 (0.97) | 0.28 (0.99) | 0.2759 | 0.0556 |
| NK.GMCSF.IgG.DBL3.7G8 | -0.09 (1.01) | 0.03 (1.05) | 0.11 (0.90) | 0.5553 | 0.7228 |
| NK.IFN.IgG.DBL3.7G8 | -0.06 (1.07) | -0.07 (0.90) | 0.24 (1.05) | 0.9675 | 0.2039 |
| NK.IL10.IgG.DBL3.7G8 | -0.08 (0.97) | 0.02 (0.97) | 0.12 (1.13) | 0.6079 | 0.6778 |
| NK.TNF.IgG.DBL3.7G8 | -0.16 (0.91) | 0.12 (1.06) | 0.06 (1.06) | 0.1626 | 0.8163 |
| IgG.DBL1.7G8 | -0.22 (1.04) | -0.01 (1.01) | 0.43 (0.77) | 0.3046 | 0.0379 |
| IgG.DBL3.7G8 | -0.18 (1.05) | 0.00 (0.80) | 0.34 (1.17) | 0.3205 | 0.1941 |
| IgG.DBL5.7G8 | -0.19 (1.14) | 0.00 (0.88) | 0.35 (0.86) | 0.3332 | 0.0978 |
| IgG.DBL6.IT4 | -0.11 (1.07) | -0.03 (0.98) | 0.26 (0.90) | 0.6748 | 0.2017 |
| IgG.DBL3.FCR3 | -0.18 (1.11) | -0.06 (0.90) | 0.44 (0.84) | 0.5581 | 0.0181 |
| IgG.DBL4.FCR3 | -0.25 (1.14) | 0.03 (0.96) | 0.40 (0.60) | 0.1874 | 0.0433 |
| IgG.DBL2(ID1-ID2a).FCR3 | -0.18 (1.12) | -0.09 (0.98) | 0.51 (0.58) | 0.6539 | 0.0013 |
| IgG.DBL4.0711 | -0.14 (1.09) | -0.04 (0.96) | 0.33 (0.84) | 0.616 | 0.0915 |
| IgG.DBL2.1010 | -0.28 (1.17) | 0.01 (0.82) | 0.50 (0.76) | 0.1537 | 0.0105 |
| IgG.DBL5.0466 | -0.17 (1.05) | -0.01 (0.86) | 0.34 (1.09) | 0.4092 | 0.1486 |
| IgG1.DBL1.7G8 | -0.08 (0.84) | 0.12 (0.78) | -0.09 (1.52) | 0.2233 | 0.5102 |
| IgG1.DBL3.7G8 | -0.02 (0.96) | -0.08 (1.01) | 0.19 (1.07) | 0.763 | 0.2847 |
| IgG1.DBL5.7G8 | -0.10 (1.03) | 0.05 (0.90) | 0.08 (1.14) | 0.4494 | 0.9322 |
| IgG1.DBL6.IT4 | -0.05 (0.89) | 0.05 (0.98) | 0.00 (1.24) | 0.5764 | 0.8473 |
| IgG1.DBL3.FCR3 | -0.05 (1.15) | -0.09 (0.91) | 0.25 (0.85) | 0.8582 | 0.1184 |
| IgG1.DBL4.FCR3 | -0.01 (1.00) | -0.01 (1.00) | 0.02 (1.04) | 0.9954 | 0.9028 |
| IgG1.DBL2(ID1-ID2a).FCR3 | -0.14 (1.07) | -0.01 (1.03) | 0.26 (0.77) | 0.5433 | 0.2079 |
| IgG1.DBL4.0711 | 0.00 (0.83) | 0.02 (0.94) | -0.04 (1.36) | 0.9429 | 0.8414 |
| IgG1.DBL2.1010 | 0.00 (0.73) | -0.01 (0.83) | 0.02 (1.59) | 0.9402 | 0.9269 |
| IgG1.DBL5.0466 | -0.07 (1.00) | 0.01 (0.91) | 0.12 (1.17) | 0.6863 | 0.6615 |
| IgG2.DBL1.7G8 | -0.12 (0.98) | 0.11 (1.08) | 0.01 (0.89) | 0.2642 | 0.639 |
| IgG2.DBL3.7G8 | -0.14 (1.04) | -0.04 (0.99) | 0.33 (0.91) | 0.6249 | 0.1052 |
| IgG2.DBL5.7G8 | -0.19 (0.79) | -0.08 (1.09) | 0.50 (1.03) | 0.5582 | 0.0247 |
| IgG2.DBL6.IT4 | 0.02 (0.94) | -0.11 (1.01) | 0.18 (1.10) | 0.5052 | 0.2581 |
| IgG2.DBL3.FCR3 | -0.11 (1.06) | 0.06 (0.99) | 0.10 (0.92) | 0.4204 | 0.8484 |
| IgG2.DBL4.FCR3 | -0.10 (0.87) | 0.01 (1.12) | 0.15 (1.00) | 0.5882 | 0.5849 |
| IgG2.DBL2(ID1-ID2a).FCR3 | -0.15 (0.81) | -0.18 (1.01) | 0.62 (1.08) | 0.84 | 0.0025 |
| IgG2.DBL4.0711 | -0.14 (0.94) | -0.12 (0.98) | 0.47 (1.03) | 0.9094 | 0.0188 |
| IgG2.DBL2.1010 | -0.18 (0.91) | -0.04 (1.10) | 0.41 (0.88) | 0.513 | 0.053 |
| IgG2.DBL5.0466 | -0.24 (0.84) | -0.11 (0.87) | 0.65 (1.22) | 0.4282 | 0.0064 |
| IgG3.DBL1.7G8 | -0.28 (0.92) | -0.02 (0.99) | 0.58 (0.96) | 0.1912 | 0.0132 |
| IgG3.DBL3.7G8 | -0.23 (1.10) | 0.05 (0.94) | 0.34 (0.81) | 0.1787 | 0.1666 |
| IgG3.DBL5.7G8 | -0.09 (1.09) | 0.01 (0.95) | 0.15 (0.95) | 0.62 | 0.5551 |
| IgG3.DBL6.IT4 | -0.14 (1.12) | -0.07 (0.92) | 0.41 (0.82) | 0.7237 | 0.0219 |
| IgG3.DBL3.FCR3 | 0.05 (0.88) | -0.25 (1.21) | 0.39 (0.58) | 0.1484 | 0.0026 |
| IgG3.DBL4.FCR3 | -0.19 (1.08) | 0.04 (1.00) | 0.28 (0.77) | 0.2667 | 0.2629 |
| IgG3.DBL2(ID1-ID2a).FCR3 | -0.15 (1.09) | -0.19 (0.92) | 0.64 (0.69) | 0.8447 | <0.0001 |
| IgG3.DBL4.0711 | -0.08 (1.01) | -0.08 (1.07) | 0.32 (0.78) | 0.994 | 0.0634 |
| IgG3.DBL2.1010 | -0.12 (1.06) | -0.14 (0.94) | 0.49 (0.88) | 0.9374 | 0.0054 |
| IgG3.DBL5.0466 | 0.00 (0.99) | -0.09 (0.95) | 0.17 (1.13) | 0.6455 | 0.3292 |
| IgG4.DBL1.7G8 | -0.15 (0.81) | -0.08 (0.91) | 0.40 (1.35) | 0.7128 | 0.1148 |
| IgG4.DBL3.7G8 | -0.19 (0.77) | -0.12 (0.82) | 0.55 (1.42) | 0.6848 | 0.0384 |
| IgG4.DBL5.7G8 | -0.19 (0.87) | -0.10 (0.69) | 0.51 (1.46) | 0.5604 | 0.0582 |
| IgG4.DBL6.IT4 | -0.11 (1.01) | -0.07 (0.85) | 0.32 (1.21) | 0.8582 | 0.1569 |
| IgG4.DBL3.FCR3 | -0.17 (0.98) | 0.00 (0.96) | 0.30 (1.08) | 0.3985 | 0.2501 |
| IgG4.DBL4.FCR3 | -0.17 (0.91) | -0.04 (0.86) | 0.37 (1.30) | 0.5089 | 0.1584 |
| IgG4.DBL2(ID1-ID2A).FCR3 | -0.18 (0.76) | -0.10 (0.90) | 0.50 (1.36) | 0.6163 | 0.0564 |
| IgG4.DBL4.0711 | 0.01 (1.06) | -0.08 (0.86) | 0.13 (1.15) | 0.6541 | 0.4418 |
| IgG4.DBL2.1010 | -0.24 (0.71) | -0.11 (0.92) | 0.61 (1.31) | 0.437 | 0.0202 |
| IgG4.DBL5.0466 | -0.05 (0.89) | -0.24 (0.78) | 0.54 (1.34) | 0.3066 | 0.0117 |
| IgA1.DBL1.7G8 | 0.14 (1.08) | -0.18 (0.89) | 0.07 (1.03) | 0.1027 | 0.2922 |
| IgA1.DBL3.7G8 | 0.10 (1.00) | -0.05 (1.03) | -0.09 (0.95) | 0.4466 | 0.8566 |
| IgA1.DBL5.7G8 | 0.07 (1.06) | -0.04 (0.93) | -0.05 (1.05) | 0.5931 | 0.9762 |
| IgA1.DBL6.IT4 | 0.15 (0.89) | -0.19 (1.03) | 0.07 (1.12) | 0.0793 | 0.325 |
| IgA1.DBL3.FCR3 | -0.02 (0.95) | -0.11 (0.94) | 0.24 (1.17) | 0.6729 | 0.1937 |
| IgA1.DBL4.FCR3 | 0.07 (0.94) | -0.08 (0.94) | 0.03 (1.22) | 0.4212 | 0.6641 |
| IgA1.DBL2(ID1-ID2A).FCR3 | 0.07 (0.96) | 0.00 (0.94) | -0.13 (1.19) | 0.6934 | 0.6237 |
| IgA1.DBL4.0711 | 0.04 (0.87) | -0.01 (1.02) | -0.06 (1.22) | 0.7953 | 0.8436 |
| IgA1.DBL2.1010 | 0.03 (1.02) | -0.02 (1.02) | -0.03 (0.97) | 0.7859 | 0.9792 |
| IgA1.DBL5.0466 | 0.04 (0.84) | 0.01 (1.15) | -0.10 (1.02) | 0.8785 | 0.6745 |
| IgA2.DBL1.7G8 | 0.16 (1.15) | -0.24 (0.92) | 0.16 (0.76) | 0.0609 | 0.0476 |
| IgA2.DBL3.7G8 | 0.09 (1.00) | -0.23 (1.00) | 0.26 (0.93) | 0.1213 | 0.0367 |
| IgA2.DBL5.7G8 | 0.10 (1.02) | -0.25 (1.00) | 0.27 (0.89) | 0.0881 | 0.0228 |
| IgA2.DBL6.IT4 | 0.11 (0.98) | -0.19 (1.01) | 0.15 (0.99) | 0.1445 | 0.1704 |
| IgA2.DBL3.FCR3 | 0.14 (1.05) | -0.25 (0.85) | 0.19 (1.10) | 0.0461 | 0.0773 |
| IgA2.DBL4.FCR3 | 0.03 (0.87) | -0.20 (1.03) | 0.31 (1.12) | 0.2258 | 0.0542 |
| IgA2.DBL2(ID1-ID2A).FCR3 | 0.11 (0.97) | -0.23 (1.06) | 0.22 (0.89) | 0.0927 | 0.0486 |
| IgA2.DBL4.0711 | 0.06 (1.00) | -0.15 (0.93) | 0.16 (1.12) | 0.2845 | 0.2376 |
| IgA2.DBL2.1010 | 0.11 (0.98) | -0.34 (0.94) | 0.41 (0.99) | 0.0212 | 0.0021 |
| IgA2.DBL5.0466 | 0.11 (0.93) | -0.17 (1.10) | 0.11 (0.92) | 0.166 | 0.2396 |
| IgM.DBL1.7G8 | 0.01 (1.00) | -0.06 (1.03) | 0.08 (0.98) | 0.7211 | 0.5678 |
| IgM.DBL3.7G8 | 0.10 (1.03) | -0.07 (1.00) | -0.07 (0.95) | 0.4017 | 0.9984 |
| IgM.DBL5.7G8 | 0.00 (1.01) | 0.02 (0.98) | -0.05 (1.06) | 0.917 | 0.7503 |
| IgM.DBL6.IT4 | 0.14 (1.02) | -0.12 (0.96) | -0.03 (1.04) | 0.1966 | 0.7186 |
| IgM.DBL3.FCR3 | -0.07 (1.08) | 0.06 (0.98) | 0.02 (0.90) | 0.5486 | 0.88 |
| IgM.DBL4.FCR3 | 0.09 (0.98) | -0.12 (1.02) | 0.05 (1.03) | 0.3041 | 0.4824 |
| IgM.DBL2(ID1-ID2a).FCR3 | 0.04 (1.05) | 0.04 (0.93) | -0.14 (1.06) | 0.9912 | 0.4826 |
| IgM.DBL4.0711 | 0.05 (1.02) | -0.11 (1.02) | 0.11 (0.94) | 0.4322 | 0.336 |
| IgM.DBL2.1010 | 0.02 (0.97) | -0.06 (0.90) | 0.07 (1.23) | 0.6601 | 0.6187 |
| IgM.DBL5.0466 | 0.02 (0.93) | -0.01 (0.97) | -0.01 (1.19) | 0.8536 | 0.9875 |
| C1q.DBL1.7G8 | -0.40 (1.29) | 0.15 (0.66) | 0.44 (0.60) | 0.0099 | 0.0579 |
| C1q.DBL3.7G8 | -0.24 (1.16) | -0.03 (0.74) | 0.49 (0.96) | 0.2787 | 0.019 |
| C1q.DBL5.7G8 | -0.29 (1.18) | 0.06 (0.90) | 0.42 (0.59) | 0.1078 | 0.0373 |
| C1q.DBL6.IT4 | -0.20 (1.17) | 0.00 (0.89) | 0.36 (0.75) | 0.3637 | 0.0625 |
| C1q.DBL3.FCR3 | -0.11 (1.06) | 0.02 (0.84) | 0.17 (1.16) | 0.5078 | 0.5453 |
| C1q.DBL4.FCR3 | -0.27 (1.01) | 0.12 (0.96) | 0.27 (0.97) | 0.0576 | 0.4963 |
| C1q.DBL2(ID1-ID2a).FCR3 | -0.24 (1.16) | -0.01 (0.92) | 0.44 (0.64) | 0.2808 | 0.0139 |
| C1q.DBL4.0711 | -0.11 (1.13) | -0.03 (1.02) | 0.25 (0.64) | 0.7252 | 0.1419 |
| C1q.DBL2.1010 | -0.20 (1.06) | -0.10 (0.99) | 0.55 (0.69) | 0.6109 | 0.0014 |
| C1q.DBL5.0466 | -0.13 (1.06) | -0.07 (1.08) | 0.38 (0.58) | 0.7816 | 0.0199 |
| FcyRI.DBL1.7G8 | -0.12 (0.97) | -0.10 (1.07) | 0.40 (0.84) | 0.8917 | 0.0274 |
| FcyRI.DBL3.7G8 | -0.15 (1.01) | -0.11 (0.96) | 0.48 (0.94) | 0.8282 | 0.0121 |
| FcyRI.DBL5.7G8 | -0.19 (1.05) | -0.02 (0.93) | 0.38 (0.95) | 0.4017 | 0.0801 |
| FcyRI.DBL6.IT4 | -0.09 (1.05) | -0.10 (1.05) | 0.36 (0.73) | 0.9375 | 0.0268 |
| FcyRI.DBL3.FCR3 | -0.12 (1.00) | -0.10 (0.94) | 0.39 (1.04) | 0.9287 | 0.0461 |
| FcyRI.DBL4.FCR3 | -0.09 (0.98) | -0.05 (1.05) | 0.26 (0.92) | 0.8294 | 0.182 |
| FcyRI.DBL2(ID1-ID2a).FCR3 | -0.22 (1.03) | -0.02 (1.00) | 0.44 (0.80) | 0.3199 | 0.0329 |
| FcyRI.DBL4.0711 | -0.06 (1.03) | -0.07 (1.05) | 0.24 (0.82) | 0.9681 | 0.1655 |
| FcyRI.DBL2.1010 | -0.16 (0.99) | -0.12 (1.03) | 0.53 (0.81) | 0.8597 | 0.0032 |
| FcyRI.DBL5.0466 | -0.16 (1.01) | -0.06 (0.95) | 0.40 (1.01) | 0.6154 | 0.0603 |
| FcyRIIA.DBL1.7G8 | -0.01 (0.97) | -0.04 (1.01) | 0.10 (1.08) | 0.9026 | 0.5974 |
| FcyRIIA.DBL3.7G8 | -0.02 (1.01) | -0.13 (0.99) | 0.28 (0.98) | 0.5944 | 0.0916 |
| FcyRIIA.DBL5.7G8 | -0.22 (1.15) | 0.08 (0.81) | 0.27 (0.95) | 0.1315 | 0.3802 |
| FcyRIIA.DBL6.IT4 | -0.20 (1.15) | 0.10 (0.90) | 0.18 (0.82) | 0.1447 | 0.7062 |
| FcyRIIA.DBL3.FCR3 | -0.03 (1.17) | -0.11 (0.84) | 0.27 (0.92) | 0.698 | 0.0769 |
| FcyRIIA.DBL4.FCR3 | -0.16 (1.08) | 0.02 (1.02) | 0.27 (0.76) | 0.3764 | 0.2367 |
| FcyRIIA.DBL2(ID1-ID2a).FCR3 | -0.19 (1.08) | -0.05 (1.01) | 0.44 (0.69) | 0.4859 | 0.0147 |
| FcyRIIA.DBL4.0711 | -0.06 (1.03) | -0.05 (1.06) | 0.21 (0.81) | 0.9442 | 0.2357 |
| FcyRIIA.DBL2.1010 | -0.18 (1.13) | -0.03 (0.89) | 0.39 (0.85) | 0.4793 | 0.0475 |
| FcyRIIA.DBL5.0466 | -0.23 (1.16) | 0.02 (0.88) | 0.39 (0.77) | 0.2437 | 0.0586 |
| FcyRIIIA.DBL1.7G8 | -0.17 (1.09) | 0.00 (0.93) | 0.31 (0.90) | 0.4104 | 0.1559 |
| FcyRIIIA.DBL3.7G8 | -0.06 (1.05) | -0.14 (0.95) | 0.37 (0.94) | 0.7003 | 0.0267 |
| FcyRIIIA.DBL5.7G8 | -0.16 (1.14) | 0.00 (0.85) | 0.30 (0.95) | 0.4201 | 0.1796 |
| FcyRIIIA.DBL6.IT4 | -0.11 (1.05) | -0.03 (1.04) | 0.26 (0.81) | 0.7275 | 0.1709 |
| FcyRIIIA.DBL3.FCR3 | 0.06 (1.06) | -0.23 (0.91) | 0.32 (0.98) | 0.1512 | 0.0214 |
| FcyRIIIA.DBL4.FCR3 | -0.14 (1.09) | -0.02 (0.99) | 0.29 (0.81) | 0.5701 | 0.1452 |
| FcyRIIIA.DBL2(ID1-ID2a).FCR3 | -0.15 (1.05) | -0.09 (1.03) | 0.43 (0.71) | 0.773 | 0.0119 |
| FcyRIIIA.DBL4.0711 | -0.13 (0.97) | -0.01 (1.14) | 0.27 (0.73) | 0.5611 | 0.1992 |
| FcyRIIIA.DBL2.1010 | -0.15 (1.08) | -0.09 (0.97) | 0.45 (0.78) | 0.7741 | 0.0104 |
| FcyRIIIA.DBL5.0466 | -0.16 (1.09) | -0.05 (0.95) | 0.39 (0.83) | 0.5834 | 0.0404 |
| FcyRIIIB.DBL1.7G8 | -0.11 (1.04) | -0.10 (0.98) | 0.39 (0.89) | 0.9299 | 0.0303 |
| FcyRIIIB.DBL3.7G8 | -0.11 (1.01) | -0.16 (0.90) | 0.49 (1.04) | 0.7979 | 0.0094 |
| FcyRIIIB.DBL5.7G8 | -0.10 (0.95) | -0.08 (0.91) | 0.32 (1.20) | 0.8944 | 0.1363 |
| FcyRIIIB.DBL6.IT4 | -0.04 (0.97) | -0.12 (1.08) | 0.29 (0.88) | 0.7057 | 0.0749 |
| FcyRIIIB.DBL3.FCR3 | -0.02 (0.98) | -0.26 (0.85) | 0.52 (1.13) | 0.193 | 0.003 |
| FcyRIIIB.DBL4.FCR3 | -0.11 (1.01) | -0.05 (1.00) | 0.31 (0.95) | 0.7608 | 0.1258 |
| FcyRIIIB.DBL2(ID1-ID2a).FCR3 | -0.17 (0.96) | -0.01 (1.02) | 0.33 (0.98) | 0.4073 | 0.1589 |
| FcyRIIIB.DBL4.0711 | -0.08 (0.98) | -0.09 (1.01) | 0.31 (0.99) | 0.9354 | 0.0977 |
| FcyRIIIB.DBL2.1010 | -0.20 (0.94) | -0.07 (0.97) | 0.51 (1.02) | 0.4964 | 0.0181 |
| FcyRIIIB.DBL5.0466 | -0.17 (1.00) | -0.06 (0.94) | 0.42 (1.02) | 0.5823 | 0.047 |
| IgG.MSP1 ^b^ | -0.17 (1.01) | 0.03 (0.96) | 0.26 (1.03) | 0.32 | 0.35 |
| IgG.SchizontExtract^b^ | 0.10 (1.06) | 0.16 (0.95) | 0.50 (0.95) | 0.76 | 0.15 |

^a^Welch’s *t*-test

^b^ Antibody feature towards non-pregnancy specific antigens (i.e not VAR2CSA), data not included in volcano plot, elastic net, or correlation networks.
