# Supplementary material for: Developing a multivariate prediction model of antibody features associated with protection of malaria-infected pregnant women from placental malaria
Source: eLife. 2021 Jun 29;10:e65776. doi: 10.7554/eLife.65776 (PMC8241440; doi:10.7554/eLife.65776)
Supplement: Supplementary file 3. [file elife-65776-supp3.docx]

**Supplementary File 3 Table: Antibody feature code, name and description**

| Code | Name | Description |
| --- | --- | --- |
| A1 | IgG1.CS2 | IgG1 levels to the surface of *Pf* infected erythrocytes (CS2 line) which express VAR2CSA |
| A2 | IgG2.CS2 | IgG2 levels to the surface of *Pf* infected erythrocytes (CS2 line) which express VAR2CSA |
| A3 | IgG3.CS2 | IgG3 levels to the surface of *Pf* infected erythrocytes (CS2 line) which express VAR2CSA |
| A4 | IgG4.CS2 | IgG4 levels to the surface of *Pf* infected erythrocytes (CS2 line) which express VAR2CSA |
| A5 | IgG.CS2 | IgG levels to the surface of *Pf* infected erythrocytes (CS2 line) which express VAR2CSA |
| A6 | IgG1.3D7 | IgG1 levels to the surface of *Pf* infected erythrocytes (3D7 line) which were selected for CSA binding |
| A7 | IgG2.3D7 | IgG2 levels to the surface of *Pf* infected erythrocytes (3D7 line) which were selected for CSA binding |
| A8 | IgG3.3D7 | IgG3 levels to the surface of *Pf* infected erythrocytes (3D7 line) which were selected for CSA binding |
| A9 | IgG4.3D7 | IgG4 levels to the surface of *Pf* infected erythrocytes (3D7 line) which were selected for CSA binding |
| A10 | IgG.3D7 | IgG levels to the surface of *Pf* infected erythrocytes (3D7 line) which were selected for CSA binding |
| B1 | THP1.Phago.CS2 | Phagocytosis of plasma opsonised *Pf* infected erythrocytes (CS2 line-which expresses VAR2CSA) by the monocyte cell line THP1 |
| B2 | THP1.Phago.IgG.CS2 | Phagocytosis of purified IgG opsonised *Pf* infected erythrocytes (CS2 line-which expresses VAR2CSA) by the monocyte cell line THP1 |
| B3 | THP1.Phago.3D7 | Phagocytosis of plasma opsonised *Pf* infected erythrocytes (3D7 line-which was selected for CSA binding) by the monocyte cell line THP1 |
| B4 | THP1.Phago.DBL2(ID1-ID2a).FCR3 | Phagocytosis of plasma opsonised beads coated with DBL2 (from *var2csa* of *Pf* line FCR3) by the monocyte cell line THP1 |
| B5 | THP1.Phago.DBL3.7G8 | Phagocytosis of plasma opsonised beads coated with DBL3 (from *var2csa* of *Pf* line 7G8) by the monocyte cell line THP1 |
| B6 | THP1.Phago.DBL5.0466 | Phagocytosis of plasma opsonised beads coated with DBL3 (from *var2csa* of a *Pf* isolate) by the monocyte cell line THP1 |
| C1 | Monocyte.Phago.CS2 | Phagocytosis of plasma opsonised *Pf* infected erythrocytes (CS2 line-which expresses VAR2CSA) by monocytes |
| C2 | Monocyte.Phago.3D7 | Phagocytosis of plasma opsonised beads coated with DBL2 (from *var2csa* of *Pf* line FCR3) by monocytes |
| C3 | Monocyte.Phago.DBL2(ID1-ID2a).FCR3 | Phagocytosis of plasma opsonised beads coated with DBL2 (from *var2csa* of *Pf* line FCR3) by monocytes |
| C4 | Monocyte.Phago.DBL3.7G8 | Phagocytosis of plasma opsonised beads coated with DBL3 (from *var2csa* of *Pf* line 7G8) by monocytes |
| C5 | Monocyte.Phago.DBL5.7G8 | Phagocytosis of plasma opsonised beads coated with DBL5 (from *var2csa* of *Pf* line 7G8) by monocytes |
| D1 | Neutrophil.ROS.DBL2.(ID1-ID2a).FCR3 | Neutrophil reactive oxygen species production to Ig binding DBL2 (from *var2csa* of *Pf* line FCR3) |
| D2 | Neutrophil.ROS.DBL3.7G8 | Neutrophil reactive oxygen species production to Ig binding DBL3 (from *var2csa* of *Pf* line 7G8) |
| D3 | Neutrophil.ROS.DBL5.7G8 | Neutrophil reactive oxygen species production to Ig binding DBL5 (from *var2csa* of *Pf* line 7G8) |
| D4 | Neutrophil.ROS.CS2 | Neutrophil reactive oxygen species production to plasma opsonised *Pf* infected erythrocytes (CS2 line-which expresses VAR2CSA) |
| D5 | Neutrophil.Phago.DBL2(ID1-ID2a).FCR3 | Phagocytosis of plasma opsonised beads coated with DBL2 (from *var2csa* of *Pf* line FCR3) by neutrophils |
| D6 | Neutrophil.Phago.DBL3.7G8 | Phagocytosis of plasma opsonised beads coated with DBL3 (from *var2csa* of *Pf* line 7G8) by neutrophils |
| D7 | Neutrophil.Phago.DBL5.7G8 | Phagocytosis of plasma opsonised beads coated with DBL5 (from *var2csa* of *Pf* line 7G8) by neutrophils |
| D8 | Neutrophil.Phago.CS2 | Phagocytosis of plasma opsonised *Pf* infected erythrocytes (CS2 line-which expresses VAR2CSA) by neutrophils |
| E1 | CSA.Binding.Inhibition.NF54 | Ability of plasma to stop *Pf* infected erythrocytes (NF54 line-selected for CSA binding) binding placental receptor CSA |
| E2 | CSA.Binding.Inhibition.FCR3 | Ability of plasma to stop *Pf* infected erythrocytes (FCR3 line-selected for CSA binding) binding placental receptor CSA |
| F1 | NK.CD107a.IgG.DBL2(ID1-ID2a).FCR3 | Expression of CD107a by NK cells in response to IgG towards DBL2 (from *var2csa* of *Pf* line FCR3) |
| F2 | NK.IFN.IgG.DBL2(ID1-ID2a).FCR3 | Expression of INFγ by NK cells in response to IgG towards DBL2 (from *var2csa* of *Pf* line FCR3) |
| F3 | NK.TNF.IgG.DBL2(ID1-ID2a).FCR3 | Expression of TNF by NK cells in response to IgG towards DBL2 (from *var2csa* of *Pf* line FCR3) |
| F4 | NK.CD107a.IgG.DBL3.7G8 | Expression of CD107a by NK cells in response to IgG towards DBL3 (from *var2csa* of *Pf* line 7G8) |
| F5 | NK.GMCSF.IgG.DBL3.7G8 | Expression of GM-CSF by NK cells in response to IgG towards DBL3 (from *var2csa* of *Pf* line 7G8) |
| F6 | NK.IFN.IgG.DBL3.7G8 | Expression of IFNγ by NK cells in response to IgG towards DBL3 (from *var2csa* of *Pf* line 7G8) |
| F7 | NK.IL10.IgG.DBL3.7G8 | Expression of IL-10 by NK cells in response to IgG towards DBL3 (from *var2csa* of *Pf* line 7G8) |
| F8 | NK.TNF.IgG.DBL3.7G8 | Expression of TNF by NK cells in response to IgG towards DBL3 (from *var2csa* of *Pf* line 7G8) |
| G1 | IgG.DBL1.7G8 | IgG levels towards DBL1 (from *var2csa* of *Pf* line 7G8) |
| G2 | IgG.DBL3.7G8 | IgG levels towards DBL3 (from *var2csa* of *Pf* line 7G8) |
| G3 | IgG.DBL5.7G8 | IgG levels towards DBL5 (from *var2csa* of *Pf* line 7G8) |
| G4 | IgG.DBL6.IT4 | IgG levels towards DBL6 (from *var2csa* of *Pf* line IT4) |
| G5 | IgG.DBL3.FCR3 | IgG levels towards DBL3 (from *var2csa* of *Pf* line FCR3) |
| G6 | IgG.DBL4.FCR3 | IgG levels towards DBL4 (from *var2csa* of *Pf* line FCR3) |
| G7 | IgG.DBL2(ID1-ID2a).FCR3 | IgG levels towards DBL2 (from *var2csa* of *Pf* line FCR3) |
| G8 | IgG.DBL4.0711 | IgG levels towards DBL4 (from *var2csa* of *Pf* isolate 0711) |
| G9 | IgG.DBL2.1010 | IgG levels towards DBL2 (from *var2csa* of *Pf* isolate 1010) |
| G10 | IgG.DBL5.0466 | IgG levels towards DBL5 (from *var2csa* of *Pf* isolate 0466) |
| H1 | IgG1.DBL1.7G8 | IgG1 levels towards DBL1 (from *var2csa* of *Pf* line 7G8) |
| H2 | IgG1.DBL3.7G8 | IgG1 levels towards DBL3 (from *var2csa* of *Pf* line 7G8) |
| H3 | IgG1.DBL5.7G8 | IgG1 levels towards DBL5 (from *var2csa* of *Pf* line 7G8) |
| H4 | IgG1.DBL6.IT4 | IgG1 levels towards DBL6 (from *var2csa* of *Pf* line IT4) |
| H5 | IgG1.DBL3.FCR3 | IgG1 levels towards DBL3 (from *var2csa* of *Pf* line FCR3) |
| H6 | IgG1.DBL4.FCR3 | IgG1 levels towards DBL4 (from *var2csa* of *Pf* line FCR3) |
| H7 | IgG1.DBL2(ID1-ID2a).FCR3 | IgG1 levels towards DBL2 (from *var2csa* of *Pf* line FCR3) |
| H8 | IgG1.DBL4.0711 | IgG1 levels towards DBL4 (from *var2csa* of *Pf* isolate 0711) |
| H9 | IgG1.DBL2.1010 | IgG1 levels towards DBL2 (from *var2csa* of *Pf* isolate 1010) |
| H10 | IgG1.DBL5.0466 | IgG1 levels towards DBL5 (from *var2csa* of *Pf* isolate 0466) |
| I1 | IgG2.DBL1.7G8 | IgG2 levels towards DBL1 (from *var2csa* of *Pf* line 7G8) |
| I2 | IgG2.DBL3.7G8 | IgG2 levels towards DBL3 (from *var2csa* of *Pf* line 7G8) |
| I3 | IgG2.DBL5.7G8 | IgG2 levels towards DBL5 (from *var2csa* of *Pf* line 7G8) |
| I4 | IgG2.DBL6.IT4 | IgG2 levels towards DBL6 (from *var2csa* of *Pf* line IT4) |
| I5 | IgG2.DBL3.FCR3 | IgG2 levels towards DBL3 (from *var2csa* of *Pf* line FCR3) |
| I6 | IgG2.DBL4.FCR3 | IgG2 levels towards DBL4 (from *var2csa* of *Pf* line FCR3) |
| I7 | IgG2.DBL2(ID1-ID2a).FCR3 | IgG2 levels towards DBL2 (from *var2csa* of *Pf* line FCR3) |
| I8 | IgG2.DBL4.0711 | IgG2 levels towards DBL4 (from *var2csa* of *Pf* isolate 0711) |
| I9 | IgG2.DBL2.1010 | IgG2 levels towards DBL2 (from *var2csa* of *Pf* isolate 1010) |
| I10 | IgG2.DBL5.0466 | IgG2 levels towards DBL5 (from *var2csa* of *Pf* isolate 0466) |
| J1 | IgG3.DBL1.7G8 | IgG3 levels towards DBL1 (from *var2csa* of *Pf* line 7G8) |
| J2 | IgG3.DBL3.7G8 | IgG3 levels towards DBL3 (from *var2csa* of *Pf* line 7G8) |
| J3 | IgG3.DBL5.7G8 | IgG3 levels towards DBL5 (from *var2csa* of *Pf* line 7G8) |
| J4 | IgG3.DBL6.IT4 | IgG3 levels towards DBL6 (from *var2csa* of *Pf* line IT4) |
| J5 | IgG3.DBL3.FCR3 | IgG3 levels towards DBL3 (from *var2csa* of *Pf* line FCR3) |
| J6 | IgG3.DBL4.FCR3 | IgG3 levels towards DBL4 (from *var2csa* of *Pf* line FCR3) |
| J7 | IgG3.DBL2(ID1-ID2a).FCR3 | IgG3 levels towards DBL2 (from *var2csa* of *Pf* line FCR3) |
| J8 | IgG3.DBL4.0711 | IgG3 levels towards DBL4 (from *var2csa* of *Pf* isolate 0711) |
| J9 | IgG3.DBL2.1010 | IgG3 levels towards DBL2 (from *var2csa* of *Pf* isolate 1010) |
| J10 | IgG3.DBL5.0466 | IgG3 levels towards DBL5 (from *var2csa* of *Pf* isolate 0466) |
| K1 | IgG4.DBL1.7G8 | IgG4 levels towards DBL1 (from *var2csa* of *Pf* line 7G8) |
| K2 | IgG4.DBL3.7G8 | IgG4 levels towards DBL3 (from *var2csa* of *Pf* line 7G8) |
| K3 | IgG4.DBL5.7G8 | IgG4 levels towards DBL5 (from *var2csa* of *Pf* line 7G8) |
| K4 | IgG4.DBL6.IT4 | IgG4 levels towards DBL6 (from *var2csa* of *Pf* line IT4) |
| K5 | IgG4.DBL3.FCR3 | IgG4 levels towards DBL3 (from *var2csa* of *Pf* line FCR3) |
| K6 | IgG4.DBL4.FCR3 | IgG4 levels towards DBL4 (from *var2csa* of *Pf* line FCR3) |
| K7 | IgG4.DBL2(ID1-ID2a).FCR3 | IgG4 levels towards DBL2 (from *var2csa* of *Pf* line FCR3) |
| K8 | IgG4.DBL4.0711 | IgG4 levels towards DBL4 (from *var2csa* of *Pf* isolate 0711) |
| K9 | IgG4.DBL2.1010 | IgG4 levels towards DBL2 (from *var2csa* of *Pf* isolate 1010) |
| K10 | IgG4.DBL5.0466 | IgG4 levels towards DBL5 (from *var2csa* of *Pf* isolate 0466) |
| L1 | IgA1.DBL1.7G8 | IgA1 levels towards DBL1 (from *var2csa* of *Pf* line 7G8) |
| L2 | IgA1.DBL3.7G8 | IgA1 levels towards DBL3 (from *var2csa* of *Pf* line 7G8) |
| L3 | IgA1.DBL5.7G8 | IgA1 levels towards DBL5 (from *var2csa* of *Pf* line 7G8) |
| L4 | IgA1.DBL6.IT4 | IgA1 levels towards DBL6 (from *var2csa* of *Pf* line IT4) |
| L5 | IgA1.DBL3.FCR3 | IgA1 levels towards DBL3 (from *var2csa* of *Pf* line FCR3) |
| L6 | IgA1.DBL4.FCR3 | IgA1 levels towards DBL4 (from *var2csa* of *Pf* line FCR3) |
| L7 | IgA1.DBL2(ID1-ID2a).FCR3 | IgA1 levels towards DBL2 (from *var2csa* of *Pf* line FCR3) |
| L8 | IgA1.DBL4.0711 | IgA1 levels towards DBL4 (from *var2csa* of *Pf* isolate 0711) |
| L9 | IgA1.DBL2.1010 | IgA1 levels towards DBL2 (from *var2csa* of *Pf* isolate 1010) |
| L10 | IgA1.DBL5.0466 | IgA1 levels towards DBL5 (from *var2csa* of *Pf* isolate 0466) |
| M1 | IgA2.DBL1.7G8 | IgA2 levels towards DBL1 (from *var2csa* of *Pf* line 7G8) |
| M2 | IgA2.DBL3.7G8 | IgA2 levels towards DBL3 (from *var2csa* of *Pf* line 7G8) |
| M3 | IgA2.DBL5.7G8 | IgA2 levels towards DBL5 (from *var2csa* of *Pf* line 7G8) |
| M4 | IgA2.DBL6.IT4 | IgA2 levels towards DBL6 (from *var2csa* of *Pf* line IT4) |
| M5 | IgA2.DBL3.FCR3 | IgA2 levels towards DBL3 (from *var2csa* of *Pf* line FCR3) |
| M6 | IgA2.DBL4.FCR3 | IgA2 levels towards DBL4 (from *var2csa* of *Pf* line FCR3) |
| M7 | IgA2.DBL2(ID1-ID2a).FCR3 | IgA2 levels towards DBL2 (from *var2csa* of *Pf* line FCR3) |
| M8 | IgA2.DBL4.0711 | IgA2 levels towards DBL4 (from *var2csa* of *Pf* isolate 0711) |
| M9 | IgA2.DBL2.1010 | IgA2 levels towards DBL2 (from *var2csa* of *Pf* isolate 1010) |
| M10 | IgA2.DBL5.0466 | IgA2 levels towards DBL5 (from *var2csa* of *Pf* isolate 0466) |
| N1 | IgM.DBL1.7G8 | IgM levels towards DBL1 (from *var2csa* of *Pf* line 7G8) |
| N2 | IgM.DBL3.7G8 | IgM levels towards DBL3 (from *var2csa* of *Pf* line 7G8) |
| N3 | IgM.DBL5.7G8 | IgM levels towards DBL5 (from *var2csa* of *Pf* line 7G8) |
| N4 | IgM.DBL6.IT4 | IgM levels towards DBL6 (from *var2csa* of *Pf* line IT4) |
| N5 | IgM.DBL3.FCR3 | IgM levels towards DBL3 (from *var2csa* of *Pf* line FCR3) |
| N6 | IgM.DBL4.FCR3 | IgM levels towards DBL4 (from *var2csa* of *Pf* line FCR3) |
| N7 | IgM.DBL2(ID1-ID2a).FCR3 | IgM levels towards DBL2 (from *var2csa* of *Pf* line FCR3) |
| N8 | IgM.DBL4.0711 | IgM levels towards DBL4 (from *var2csa* of *Pf* isolate 0711) |
| N9 | IgM.DBL2.1010 | IgM levels towards DBL2 (from *var2csa* of *Pf* isolate 1010) |
| N10 | IgM.DBL5.0466 | IgM levels towards DBL5 (from *var2csa* of *Pf* isolate 0466) |
| O1 | C1q.DBL1.7G8 | C1q binding of Ig towards DBL1 (from *var2csa* of *Pf* line 7G8) |
| O2 | C1q.DBL3.7G8 | C1q binding of Ig towards DBL3 (from *var2csa* of *Pf* line 7G8) |
| O3 | C1q.DBL5.7G8 | C1q binding of Ig towards DBL5 (from *var2csa* of *Pf* line 7G8) |
| O4 | C1q.DBL6.IT4 | C1q binding of Ig towards DBL6 (from *var2csa* of *Pf* line IT4) |
| O5 | C1q.DBL3.FCR3 | C1q binding of Ig towards DBL3 (from *var2csa* of *Pf* line FCR3) |
| O6 | C1q.DBL4.FCR3 | C1q binding of Ig towards DBL4 (from *var2csa* of *Pf* line FCR3) |
| O7 | C1q.DBL2(ID1-ID2a).FCR3 | C1q binding of Ig towards DBL2 (from *var2csa* of *Pf* line FCR3) |
| O8 | C1q.DBL4.0711 | C1q binding of Ig towards DBL4 (from *var2csa* of *Pf* isolate 0711) |
| O9 | C1q.DBL2.1010 | C1q binding of Ig towards DBL2 (from *var2csa* of *Pf* isolate 1010) |
| O10 | C1q.DBL5.0466 | C1q binding of Ig towards DBL5 (from *var2csa* of *Pf* isolate 0466) |
| P1 | FcyRI.DBL1.7G8 | FcγRI binding of Ig towards DBL1 (from *var2csa* of *Pf* line 7G8) |
| P2 | FcyRI.DBL3.7G8 | FcγRI binding of Ig towards DBL3 (from *var2csa* of *Pf* line 7G8) |
| P3 | FcyRI.DBL5.7G8 | FcγRI binding of Ig towards DBL5 (from *var2csa* of *Pf* line 7G8) |
| P4 | FcyRI.DBL6.IT4 | FcγRI binding of Ig towards DBL6 (from *var2csa* of *Pf* line IT4) |
| P5 | FcyRI.DBL3.FCR3 | FcγRI binding of Ig towards DBL3 (from *var2csa* of *Pf* line FCR3) |
| P6 | FcyRI.DBL4.FCR3 | FcγRI binding of Ig towards DBL4 (from *var2csa* of *Pf* line FCR3) |
| P7 | FcyRI.DBL2(ID1-ID2a).FCR3 | FcγRI binding of Ig towards DBL2 (from *var2csa* of *Pf* line FCR3) |
| P8 | FcyRI.DBL4.0711 | FcγRI binding of Ig towards DBL4 (from *var2csa* of *Pf* isolate 0711) |
| P9 | FcyRI.DBL2.1010 | FcγRI binding of Ig towards DBL2 (from *var2csa* of *Pf* isolate 1010) |
| P10 | FcyRI.DBL5.0466 | FcγRI binding of Ig towards DBL5 (from *var2csa* of *Pf* isolate 0466) |
| Q1 | FcyRIIA.DBL1.7G8 | FcγRIIA binding of Ig towards DBL1 (from *var2csa* of *Pf* line 7G8) |
| Q2 | FcyRIIA.DBL3.7G8 | FcγRIIA binding of Ig towards DBL3 (from *var2csa* of *Pf* line 7G8) |
| Q3 | FcyRIIA.DBL5.7G8 | FcγRIIA binding of Ig towards DBL5 (from *var2csa* of *Pf* line 7G8) |
| Q4 | FcyRIIA.DBL6.IT4 | FcγRIIA binding of Ig towards DBL6 (from *var2csa* of *Pf* line IT4) |
| Q5 | FcyRIIA.DBL3.FCR3 | FcγRIIA binding of Ig towards DBL3 (from *var2csa* of *Pf* line FCR3) |
| Q6 | FcyRIIA.DBL4.FCR3 | FcγRIIA binding of Ig towards DBL4 (from *var2csa* of *Pf* line FCR3) |
| Q7 | FcyRIIA.DBL2(ID1-ID2a).FCR3 | FcγRIIA binding of Ig towards DBL2 (from *var2csa* of *Pf* line FCR3) |
| Q8 | FcyRIIA.DBL4.0711 | FcγRIIA binding of Ig towards DBL4 (from *var2csa* of *Pf* isolate 0711) |
| Q9 | FcyRIIA.DBL2.1010 | FcγRIIA binding of Ig towards DBL2 (from *var2csa* of *Pf* isolate 1010) |
| Q10 | FcyRIIA.DBL5.0466 | FcγRIIA binding of Ig towards DBL5 (from *var2csa* of *Pf* isolate 0466) |
| R1 | FcyRIIIA.DBL1.7G8 | FcγRIIIA binding of Ig towards DBL1 (from *var2csa* of *Pf* line 7G8) |
| R2 | FcyRIIIA.DBL3.7G8 | FcγRIIIA binding of Ig towards DBL3 (from *var2csa* of *Pf* line 7G8) |
| R3 | FcyRIIIA.DBL5.7G8 | FcγRIIIA binding of Ig towards DBL5 (from *var2csa* of *Pf* line 7G8) |
| R4 | FcyRIIIA.DBL6.IT4 | FcγRIIIA binding of Ig towards DBL6 (from *var2csa* of *Pf* line IT4) |
| R5 | FcyRIIIA.DBL3.FCR3 | FcγRIIIA binding of Ig towards DBL3 (from *var2csa* of *Pf* line FCR3) |
| R6 | FcyRIIIA.DBL4.FCR3 | FcγRIIIA binding of Ig towards DBL4 (from *var2csa* of *Pf* line FCR3) |
| R7 | FcyRIIIA.DBL2(ID1-ID2a).FCR3 | FcγRIIIA binding of Ig towards DBL2 (from *var2csa* of *Pf* line FCR3) |
| R8 | FcyRIIIA.DBL4.0711 | FcγRIIIA binding of Ig towards DBL4 (from *var2csa* of *Pf* isolate 0711) |
| R9 | FcyRIIIA.DBL2.1010 | FcγRIIIA binding of Ig towards DBL2 (from *var2csa* of *Pf* isolate 1010) |
| R10 | FcyRIIIA.DBL5.0466 | FcγRIIIA binding of Ig towards DBL5 (from *var2csa* of *Pf* isolate 0466) |
| S1 | FcyRIIIB.DBL1.7G8 | FcγRIIIB binding of Ig towards DBL1 (from *var2csa* of *Pf* line 7G8) |
| S2 | FcyRIIIB.DBL3.7G8 | FcγRIIIB binding of Ig towards DBL3 (from *var2csa* of *Pf* line 7G8) |
| S3 | FcyRIIIB.DBL5.7G8 | FcγRIIIB binding of Ig towards DBL5 (from *var2csa* of *Pf* line 7G8) |
| S4 | FcyRIIIB.DBL6.IT4 | FcγRIIIB binding of Ig towards DBL6 (from *var2csa* of *Pf* line IT4) |
| S5 | FcyRIIIB.DBL3.FCR3 | FcγRIIIB binding of Ig towards DBL3 (from *var2csa* of *Pf* line FCR3) |
| S6 | FcyRIIIB.DBL4.FCR3 | FcγRIIIB binding of Ig towards DBL4 (from *var2csa* of *Pf* line FCR3) |
| S7 | FcyRIIIB.DBL2(ID1-ID2a).FCR3 | FcγRIIIB binding of Ig towards DBL2 (from *var2csa* of *Pf* line FCR3) |
| S8 | FcyRIIIB.DBL4.0711 | FcγRIIIB binding of Ig towards DBL4 (from *var2csa* of *Pf* isolate 0711) |
| S9 | FcyRIIIB.DBL2.1010 | FcγRIIIB binding of Ig towards DBL2 (from *var2csa* of *Pf* isolate 1010) |
| S10 | FcyRIIIB.DBL5.0466 | FcγRIIIB binding of Ig towards DBL5 (from *var2csa* of *Pf* isolate 0466) |
