# Supplementary material for: Developing a multivariate prediction model of antibody features associated with protection of malaria-infected pregnant women from placental malaria
Source: eLife. 2021 Jun 29;10:e65776. doi: 10.7554/eLife.65776 (PMC8241440; doi:10.7554/eLife.65776)
Supplement: Supplementary file 4. [file elife-65776-supp4.docx]

**Supplementary File 4-table 1: Association between selected antibody features and gravidity^a^ in 77 women (women with placental malaria or non-placental infection at delivery)**

| Antibody feature | Coefficient | 95% CI | p-value |
| --- | --- | --- | --- |
| IgG3.DBL2(ID1-ID2a).FCR3 | 0.0007 | -0.25, 0.25 | 1.00 |
| THP1.Phago.CS2 | 0.19 | -0.07, 0.45 | 0.15 |
| Neutrophil.Phago.CS2 | 0.2 | -0.06, 0.46 | 0.13 |
| CSA.Binding.Inhibition.FCR3 | 0.3 | 0.005, 0.59 | 0.046 |
| IgA2.DBL2.1010 | 0.038 | -0.23, 0.30 | 0.78 |
| IgG3.3D7 | -0.04 | -0.31, 0.23 | 0.78 |

^a^Women were classified as primigravidae, 1; secundigravidae, 2; or multigravidae, 3.

**Supplementary File 4-table 2: Univariate analysis of selected antibody features placental malaria v non-placental infection, in women uninfected at enrolment^a^**

| Antibody feature | Placental malaria  (n=21) | Non-placental infection  (n=43) | Placental malaria v Non-placental infection |
| --- | --- | --- | --- |
|  | mean (SD) | mean (SD) | p value |
| IgG3.DBL2(ID1-ID2a).FCR3 | -0.25 (0.87) | 0.55 (0.62) | <0.000 |
| THP1.Phago.CS2 | -0.28 (0.80) | 0.47 (1.04) | 0.002 |
| Neutrophil.Phago.CS2 | -0.21 (1.06) | 0.46 (0.56) | 0.009 |
| CSA.Binding.Inhibition.FCR3 | -0.28 (1.39) | 0.31 (0.43) | 0.064 |
| IgA2.DBL2.1010 | -0.28 (0.98) | 0.40 (1.03) | 0.013 |
| IgG3.3D7 | -.12 (0.89) | 0.36 (1.05) | 0.062 |

^a^Women who had *P. falciparum* infection at enrolment by PCR and/or light microscopy were excluded from this analysis.
