# Supplementary material for: Developing a multivariate prediction model of antibody features associated with protection of malaria-infected pregnant women from placental malaria
Source: eLife. 2021 Jun 29;10:e65776. doi: 10.7554/eLife.65776 (PMC8241440; doi:10.7554/eLife.65776)
Supplement: Transparent reporting form [file elife-65776-transrepform.docx]

***eLife’s* transparent reporting form**

We encourage authors to provide detailed information *within their submission* to facilitate the interpretation and replication of experiments. Authors can upload supporting documentation to indicate the use of appropriate reporting guidelines for health-related research (see [EQUATOR Network](http://www.equator-network.org/%20)), life science research (see the [BioSharing Information Resource](https://biosharing.org/)), or the [ARRIVE guidelines](http://www.plosbiology.org/article/info:doi/10.1371/journal.pbio.1000412) for reporting work involving animal research. Where applicable, authors should refer to any relevant reporting standards documents in this form.

If you have any questions, please consult our Journal Policies and/or contact us: [editorial@elifesciences.org](mailto:editorial@elifesciences.org).

**Sample-size estimation**

- You should state whether an appropriate sample size was computed when the study was being designed
- You should state the statistical method of sample size computation and any required assumptions
- If no explicit power analysis was used, you should describe how you decided what sample (replicate) size (number) to use

No explicit power analysis was used and therefore was not included in the manuscript. The study did not aim to identify a difference between cohorts but rather feature/s which contribute to differences. Sample size was based on published articles which have used a similar approach (systems serology) to investigate antibody responses to other infectious diseases. For one of the groups (non-placental infection) the sample size was also limited on sample availability as few women met the criteria for inclusion.

Please outline where this information can be found within the submission (e.g., sections or figure legends), or explain why this information doesn’t apply to your submission:

**Replicates**

- You should report how often each experiment was performed
- You should include a definition of biological versus technical replication
- The data obtained should be provided and sufficient information should be provided to indicate the number of independent biological and/or technical replicates
- If you encountered any outliers, you should describe how these were handled
- Criteria for exclusion/inclusion of data should be clearly stated
- High-throughput sequence data should be uploaded before submission, with a private link for reviewers provided (these are available from both GEO and ArrayExpress)

Please outline where this information can be found within the submission (e.g., sections or figure legends), or explain why this information doesn’t apply to your submission:

All antibody feature measurements made with primary cells (neutrophils, NK cells and monocytes)- were repeated with three different primary cell donors an average of the three used.

This information is found under the appropriate headings in the methods sections as detailed below.

## **Method Details**

**ADCP of VAR2CSA DBL-coated Beads by Monocytes**

### **ADCP of IE by Monocytes**

### **ADNP of VAR2CSA DBL-coated Beads**

### **ADNP of IE**

**ADRB using VAR2CSA DBL Domains**

### **ADRB to IE**

**ADCC using VAR2CSA DBL Domains**

All antibody feature measurements except for the neutrophil ROS, multiplex and NK cell assays were done in duplicate (note- neutrophil ROS and NK cell assays were still repeated with three donors- as detailed above).

This information is found under the appropriate headings in the methods sections as detailed below.

## **Method Details**

**Antibody Features to VAR2CSA by Multiplex**

### **Detection of Antibody binding to the IE**

### **ADCP of VAR2CSA DBL-coated Beads by THP-1**

### **ADCP of IE by THP-1**

**ADCP of VAR2CSA DBL-coated Beads by Monocytes**

### **ADCP of IE by Monocytes**

### **ADNP of VAR2CSA DBL-coated Beads**

### **ADNP of IE**

**ADRB using VAR2CSA DBL Domains**

### **ADRB to IE**

### **CSA Binding Inhibition Antibody to IE**

All analysis was performed on a single cohort, this information is included in methods section under “**Human Subjects**”.

### Repeated analysis on resampled data is described in the methods section under “**Identification of Key Antibody Features”.**

Outliers were not excluded from analysis.

### Details of samples and data included in all stages of the analysis is clearly described in **Quantification and Statistical Analysis** and **Figure 1-figure supplement 2**

Excluded data- no data was excluded prior to statistical analysis except for certain multiplex data points. The criteria for this is detailed in the sections below.

**Method Details**

### **Antibody Features to VAR2CSA by Multiplex**

**Statistical reporting**

- Statistical analysis methods should be described and justified
- Raw data should be presented in figures whenever informative to do so (typically when N per group is less than 10)
- For each experiment, you should identify the statistical tests used, exact values of N, definitions of center, methods of multiple test correction, and dispersion and precision measures (e.g., mean, median, SD, SEM, confidence intervals; and, for the major substantive results, a measure of effect size (e.g., Pearson's r, Cohen's d)
- Report exact p-values wherever possible alongside the summary statistics and 95% confidence intervals. These should be reported for all key questions and not only when the p-value is less than 0.05.

Please outline where this information can be found within the submission (e.g., sections or figure legends), or explain why this information doesn’t apply to your submission:

Statistical analysis is described in detail in the methods sections under the headings

## **Quantification and Statistical Analysis**

### **Processing of Data**

### **Univariate analysis**

### **Identification of Key antibody features**

Statistical tests are all identified, exact values of N are specified (in methods section under “**Human Subjects**”), exact P values are stated for all questions where applicable.

In the univariate analysis a large number of statistical tests were run (figure 1) and so we have included a supplementary table (**supplementary File 2**) of mean (SD) and P for all the tests.

(For large datasets, or papers with a very large number of statistical tests, you may upload a single table file with tests, Ns, etc., with reference to sections in the manuscript.)

**Group allocation**

- Indicate how samples were allocated into experimental groups (in the case of clinical studies, please specify allocation to treatment method); if randomization was used, please also state if restricted randomization was applied
- Indicate if masking was used during group allocation, data collection and/or data analysis

Please outline where this information can be found within the submission (e.g., sections or figure legends), or explain why this information doesn’t apply to your submission:

Detailed description of sample selection and allocation into the case control study groups is in the methods section under “**Human Subjects**”.

**Additional data files (“source data”)**

- We encourage you to upload relevant additional data files, such as numerical data that are represented as a graph in a figure, or as a summary table
- Where provided, these should be in the most useful format, and they can be uploaded as “Source data” files linked to a main figure or table
- Include model definition files including the full list of parameters used
- Include code used for data analysis (e.g., R, MatLab)
- Avoid stating that data files are “available upon request”

Please indicate the figures or tables for which source data files have been provided:

We uploaded source data for **Figure 3,** all antibody feature measurements used in the analysis **(Figures 1-5)** have been uploaded onto Datadryad.

Code used for data analysis has been clearly described with references in the methods section under “**Quantification and Statistical Analysis”.**

The full list and description of parameters used is listed in **supplementary file 3**
